# Quantitative Raman Spectroscopy Analysis of Polyhydroxyalkanoates Produced by *Cupriavidus necator* H16

**DOI:** 10.3390/s16111808

**Published:** 2016-10-28

**Authors:** Ota Samek, Stanislav Obruča, Martin Šiler, Petr Sedláček, Pavla Benešová, Dan Kučera, Ivana Márova, Jan Ježek, Silva Bernatová, Pavel Zemánek

**Affiliations:** 1 Institute of Scientific Instruments of the CAS, Brno 61264, Czech Republic; siler@isibrno.cz (M.S.); jezek@isibrno.cz (J.J.); berns@isibrno.cz (S.B.); pavlik@isibrno.cz (P.Z.); 2 Materials Research Centre, Faculty of Chemistry, Brno University of Technology, Brno 61200, Czech Republic; Stana.O@seznam.cz (S.O.); sedlacek-p@fch.vutbr.cz (P.S.); xcbenesova@fch.vut.cz (P.B.); xckucera@fch.vut.cz (D.K.); marova@fch.vut.cz (I.M.)

**Keywords:** Raman spectroscopy, *Cupriavidus necator* H16, polyhydroxyalkanoates

## Abstract

We report herein on the application of Raman spectroscopy to the rapid quantitative analysis of polyhydroxyalkanoates (PHAs), biodegradable polyesters accumulated by various bacteria. This theme was exemplified for quantitative detection of the most common member of PHAs, poly(3-hydroxybutyrate) (PHB) in *Cupriavidus necator* H16. We have identified the relevant spectral region (800–1800 cm^−1^) incorporating the Raman emission lines exploited for the calibration of PHB (PHB line at 1736 cm^−1^) and for the selection of the two internal standards (DNA at 786 cm^−1^ and Amide I at 1662 cm^−1^). In order to obtain quantitative data for calibration of intracellular content of PHB in bacterial cells reference samples containing PHB amounts—determined by gas chromatography—from 12% to 90% (w/w) were used. Consequently, analytical results based on this calibration can be used for fast and reliable determination of intracellular PHB content during biotechnological production of PHB since the whole procedure—from bacteria sampling, centrifugation, and sample preparation to Raman analysis—can take about 12 min. In contrast, gas chromatography analysis takes approximately 8 h.

## 1. Introduction

PHAs are polyesters of natural origin accumulated as carbon and energy storage materials in a form of intracellular granules by a wide variety of bacterial strains including Gram-negative and Gram-positive species (i.e., autotrophic, heterotrophic, and phototrophic microorganisms, aerobes and anaerobes) as well as for some Archae strains. Due to their mechanical properties resembling synthetic polymers and their fully biodegradable and biocompatible nature, PHAs are considered being ecologically friendly alternative for petrochemical plastics [1,2]. Nevertheless, there are several aspects that prevent PHAs from massively entering the market. The cost of PHAs is significantly higher than that of synthetic polymers. Since about 45% of the total costs of PHA production are ascribed to carbon sources, such as refined glucose or sucrose. Therefore, cheap wastes or side products of agriculture and food industry, are used as inexpensive carbon substrates, improving thus the economic feasibility of the PHA production [2,3]. Further, the biotechnological production of PHAs is also complicated by the lack of fast and reliable analytical tools enabling the rapid and sensitive determination of PHAs in bacterial cells during the biotechnological process. The most commonly employed method for quantitative analysis of PHAs is gas chromatography; nevertheless, the labor intensity and time demands of this method prevents its application for routine analysis of PHAs during the biotechnological process. Apart from gas chromatography, there are other techniques (such as gravimetry, turbidimetry, UV spectroscopy, optical fluorescence, and electron microscopy) that can be used for the determination of PHAs in bacterial cells [4], but most of them are time consuming or do not provide sufficient reliability or sensitivity to be used in the PHA production process control.

Therefore, the main aim of our study was to utilize Raman spectroscopy for the quantitative determination of the most common member of PHAs family, poly(3-hydroxybutyrate) (PHB). For this purpose, cells with various contents of PHB are referenced to selected internal standards using dedicated Raman spectra of bacterial strain *Cupriavidus necator* H16, which is considered a model microorganism for PHA metabolism and a valid candidate for the industrial production of PHAs.

The technique of Raman spectroscopy is slowly becoming a well recognized analytical technique because of the identification of its competitive position amongst other techniques [5,6,7]. Raman spectroscopy has an extremely competitive position if in situ, contactless, noninvasive, and fast analysis is required, say, on-line in biotechnological processes. The sample does not have to be prepared for analysis using solvents; the technique is label free with minimal interference from water [8,9,10,11,12,13,14,15,16,17,18,19].

For the analysis of PHB-producing bacteria, experiments have recently been performed in which PHB spectra were recognized [19,20]. Additionally, studies using the IR-Raman technique were performed on different polymers [21,22].

However, studies [19,20] do not provide reliable quantitative analysis over the entire range of PHB concentrations required by biotechnological production. In [19], the authors used Raman band intensities plotted against PHB content determined by HPLC. The highest concentration of PHB was about 0.2 g/L (note that industrial applications require concentrations of about tens of g/L). *Cupriavidus necator* H16 has been used recently to monitor the PHB fermentation process [23] using the intensities of the Raman bands.

In contrast, our work presents Raman intensity ratios that, rather than simply use the spectral intensity, were recorded and used to construct a calibration curve. This process is often referred to as internal standardization and eliminates different experimental factors such as laser power and instrumental effects. Recently, internal standardization employing Raman spectroscopy was used for the rapid determination of the quality of turmeric roots [24].

Thus, our study presents full calibration curves based on internal standards covering a large range of PHB concentrations—up to 90% (w/w). To the best of our knowledge, such data has not been published previously. Our results are convincing, and we believe that our study will be of significant assistance to research groups being involved in the biotechnological production of PHB.

## 2. Materials and Methods

### 2.1. Microorganisms and Their Cultivation

*Cupriavidus necator* H16 (CCM 3726) was obtained from the Czech Collection of Microorganisms (Masaryk University, Faculty of Science, Brno, Czech Republic).

To obtain cells with various amounts of intracellular content of PHB, a culture of *Cupriavidus necator* H16 was cultivated in Erlenmeyer flasks (volume 250 mL) containing 100 mL of a mineral salt (MS) medium described elsewhere [25] containing various concentrations of fructose (5–20 g/L) as a sole carbon source and (NH_4_)_2_SO_4_ (1–5 g/L) as a sole nitrogen source. The flasks were inoculated by 5 mL of an overnight culture of cells grown in a nutrient broth (NB) medium (NB medium: 10 g of peptone, 10 g of beef extract, and 5 g of NaCl in 1 L of distilled water). The samples were taken at various cultivation times, the cells were harvested (centrifugation, 8000× *g*, 5 min), and PHB content was analyzed via gas chromatography and Raman spectroscopy.

Before PHB analysis via gas chromatography, the cells washed with 5% (vol/vol) Triton X (10 mL) and distilled water and dried overnight at 105 °C. Consequently, the PHB content of dried cells was analyzed via gas chromatography (Trace GC Ultra, Thermo Scientific, Waltham, MA, USA). Commercially available PHB (Sigma Aldrich, Germany) was used as a standard; benzoic acid (LachNer, Neratovice, Czech Republic) was used as an internal standard.

### 2.2. Bacterial Sample Preparation and Estimated Time Frame for Analysis

To prepare samples for Raman analysis, the following steps were addressed:

(a) One milliliter of bacterial culture was transferred into a 1.5 mL tube, and the cells were centrifuged (10,000 rpm, 2 min), washed with 50% ethanol, and centrifuged again (total 5 min).
(b) In the next step, the supernatant was aspirated, and approx. 20 µL of cell pellets formed via centrifugation was pipetted onto a CaF (Raman grade) microscope slide (1 min).
(c) The cell suspensions were air-dried (approx. 5 min) at laboratory temperature and analyzed using a Raman instrument (1 min).

Thus, the entire procedure of sample preparation and analysis takes about 12 min, while that of GC technique analysis takes approximately 8 h. Note that the air drying step can be skipped so that only the pipetted sample can be measured. We found that focusing on the dried sample is easier, so that is why this step was also included in the above procedure.

### 2.3 Raman Spectroscopy

Cells were analyzed using a Renishaw Invia system (Renishaw inVia Raman Spectrometer, Renishaw plc., Wotton under Edge, UK), with a 785 nm single-mode diode laser as the excitation source. A laser beam was focused onto a sample by the microscope objective (50×, NA 0.5, Leica, Wetzlar, Germany) with a laser spot diameter of approximately 2 μm × 10 μm, which is a characteristic feature for this type of Raman spectroscopy instrument supplied by Renishaw. Overview spectra were acquired in the range of 700–1800 cm^−1^. Each spectrum used for calibration was measured for 15 s in a total of 3 measurements for one sample.

### 2.4. Data Analysisis

The Raman spectra were treated with the Savitzky–Golay procedure coupled with an advanced rolling filter background removal routine (see [12]), normalized to Amide I (1656 cm^−1^) or DNA (878 cm^−1^); subsequently, emission line intensities of interest were estimated and averaged for the construction of the calibration plot. The program written in-house using MatLab software (MathWorks, Natick, MA, USA) was used. Both Raman features used for normalization are present in the tested microorganisms and were used for internal standardization after following numerous tests exploring the reliability of these internal standards.

## 3. Results and Discussion

**Figure 1:**
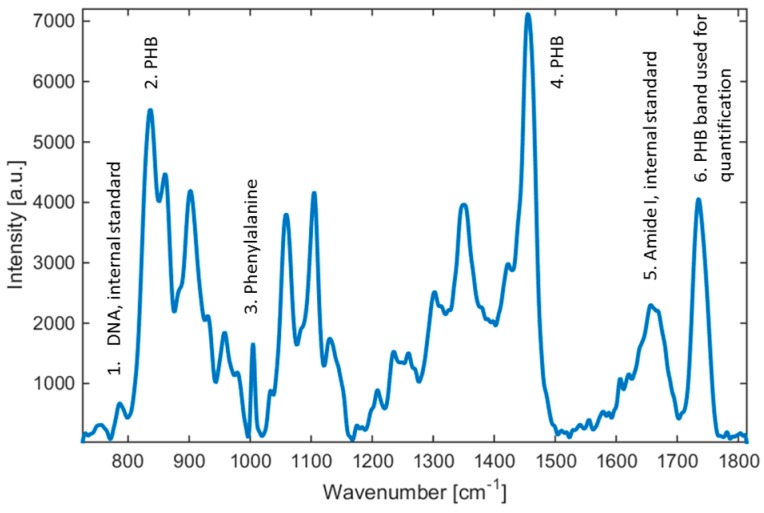
Raman spectra of *Cupriavidus necator* H16. Selected emission lines used in our study are highlighted. Note that peaks 1, 5, and 6 can be used for analysis of PHB in the bacteria sample—peak 6 represents the PHB band used in this study, peak 5 is an internal standard (Amide I), and peak 1 is a second internal standard (DNA).

Figure 1 shows a typical Raman spectrum, here for *Cupriavidus necator* H16 cultured directly on a Petri dish. It should be pointed out that, for the sake of simplicity, the samples can be also analyzed directly on the Petri dishes on which the bacteria were cultivated.

As can be seen from Figure 1, the whole cell Raman spectrum revealed some characteristic emission lines which can be attributed to common cell components such as DNA (phosphodiester bond at 786 cm^−1^), proteins (phenylalanine at 1005 cm^−1^), Amide I (1662 cm^−1^), and lipids (C–H vibrations at 1449 cm^−1^ or 1301 cm^−1^). PHB provides several Raman peaks (837, 1455, and 1736 cm^−1^) that can be used for PHB identification and further analysis. Among them, the emission lines at 1736 cm^−1^ were selected and can be considered to be the most useful candidate because the vibrations of the other components of bacterial cells do not interfere with this emission line.

**Figure 2:**
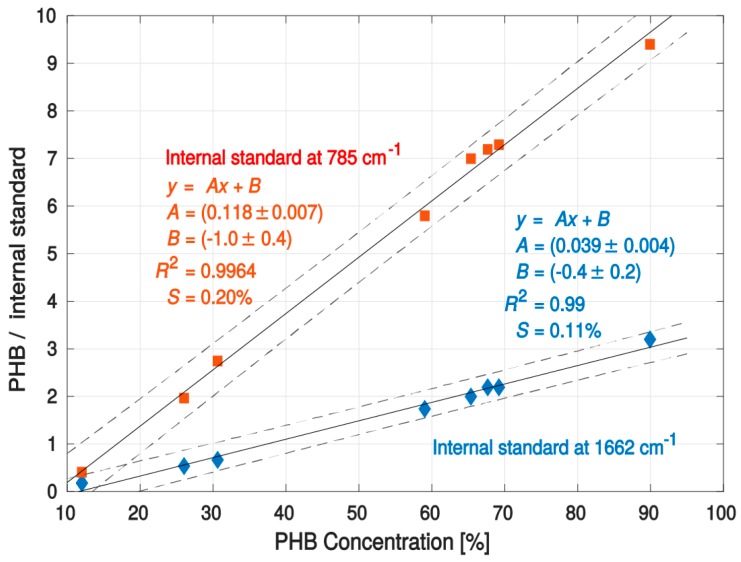
Calibration plot for PHB in *Cupriavidus necator* over a PHB concentration range of 12%–90% (by mass). The upper calibration curve (red squares) emission line at 787 cm^−1^ (DNA) was used as an internal standard. The bottom calibration curve was constructed using a line at 1662 cm^−1^ (Amide I) as an internal standard. The dashed curves show a 95% confidence band of linear fit. Parameters describing the calibration curves are shown in the figure (including 95% error interval). R^2^ is the coefficient of determination. *S* is the mean square error. The calibration curve provides an excellent fit to the data over the region of interest. Normalized intensities of emission lines used for calibration and cultivation conditions and media used for growing bacteria are listed in Table 1.

For the calibration, reference samples of various PHB contents were prepared and the exact amounts of PHB in these samples were determined using an established method of gas chromatography (GC). Reference samples with PHB concentrations in the range from 12% to 90% (w/w) were used, as detailed in Table 1. Since it can be expected that intracellular concentration of proteins and nucleic acid is more or less constant with varying intracellular PHB concentration, line intensities at 787 cm^−1^ (DNA) and at 1662 cm^−1^ (Amide I) were proposed as internal standards for a reliable calibration procedure (Figure 1). Thus, the acquired Raman spectra of the PHB line were normalized to one of these values (see Table 1). Consequently, the two resulting calibration curves are shown in Figure 2.

In our experiments, we focused on bacteria (*Cupriavidus necator* H16) that are a model producer of PHB. Ideally, more reference samples should be used to construct a calibration curve. During this work, however, only eight referenced samples covering concentrations of interest for biotechnological production (with PHB determined by gas chromatography) were available.

The calibration curves clearly show an excellent linear fit to the data over the region of interest. It can be seen that all measured data points can be found close to the center of the 95% confidence bounds. In order to validate or justify our calibration set and curves, we performed a cross-validation based on the “leave-one-out” cross-validation method. The slopes of lines fitted to the training sets corresponded with the presented experimental results for the full data sets. Moreover, the root mean square error taken from all validation points was significantly smaller than the width of the presented 95% confidence band. This demonstrates the statistical significance of the data. Therefore, we believe that the presented results are based on solid data, and the calibration curve can be readily used in different biotechnological applications, exploiting PHB amounts in *Cupriavidus necator* H16 during the cultivation process.

It should be noted that the calibration curves are limited only for the amorphous state of the polymer found inside the cells. We noticed that only in a few cases (most likely depending on incorrect treatment of cells before analysis) could the amorphous state be changed such that crystallization appears as a great enhancement of intensity or shift of PHB-corresponding Raman peaks [26]. This can be favorably solved—the qualitative difference between crystalline and amorphous polymer states can be evaluated by simply monitoring the position of the PHB line of interest (peak 6 in Figure 1) within the spectra. In the case of crystallization, this band may vary up to 15 cm^−1^ (from 1736 cm^−1^ to about 1721 cm^−1^) in the Raman spectra of bacteria, which is a clear sign that the crystallization will occur and can be readily controlled by the naked eye.

We used a univariate approach because it is simple to use. Indeed, we have tried multivariate approaches, however, we have not obtained any reasonable improvement of our results. Note that, for other PHB-producing bacterial strains, we considered that the amount of proteins and DNA (which we used as internal standards) is different for different species producing PHB. Thus, our calibration curve should not be used as a generic tool for the quantification of PHB in different PHB-producing strains.

## 4. Conclusions

In summary, we have herein demonstrated an easy-to-apply Raman spectroscopy technique for the qualitative and quantitative determination of the amount of poly(3-hydroxybutyrate) (PHB) in bacteria *Cupriavidus necator* H16. The technique is applicable to near real-time and in-situ monitoring at the process control of PHB bio production. Here, Raman spectroscopy demonstrates clear benefits over existing chemical means of identification, such as gas chromatography in the speed and noninvasivity of the quantification process.

## Figures and Tables

**Table 1:** Data used for the construction of calibration curves shown in Figure 2. PHB reference values were estimated using the GC technique. Cultivation conditions for each reference sample are also provided.

Cultivation Conditions	PHB %	Intensity of PHB Line at 1736 cm^−1^ (Normalized on Amide I at 1662 cm^–1^)	Intensity of PHB Line at 1736 cm^–1^ (Normalized on DNA at 785 cm^–1^)
*Cupriavidus necator* H16	12.1	0.17	0.39
MS medium, 5 g/L fructose, 1 g/L (NH_4_)_2_SO_4_, from Petri dish, 72 h of cultivation			
*Cupriavidus necator* H16	26.1	0.55	2.0
MS medium, 5 g/L fructose, 3 g/L (NH_4_)_2_SO_4_, 72 h of cultivation			
*Cupriavidus necator* H16	30.6	0.65	2.7
NB medium, 24 h of cultivation			
*Cupriavidus necator* H16	59.1	1.8	5.8
MS medium, 20 g/L fructose, 1 g/L (NH_4_)_2_SO_4_, 24 h of cultivation			
*Cupriavidus necator* H16	65.4	2.0	7.0
MS medium, 20 g/L fructose, 3 g/L (NH_4_)_2_SO_4_, 24 h of cultivation			
*Cupriavidus necator* H16	67.7	2.2	7.2
MS medium, 20 g/L fructose, 3 g/l (NH_4_)_2_SO_4_, 24 h of cultivation			
*Cupriavidus necator* H16	69.2	2.2	7.3
MS medium, 20 g/L fructose, 3 g/L (NH_4_)_2_SO_4_, 72 h of cultivation			
*Cupriavidus necator* H16	90.0	3.2	9.4
MS medium, 20 g/L fructose, 3 g/L (NH_4_)_2_SO_4_, 72 h of cultivation			
